# Fancy-Shaped Gold–Platinum Nanocauliflowers for Improved Proton Irradiation Effect on Colon Cancer Cells

**DOI:** 10.3390/ijms21249610

**Published:** 2020-12-17

**Authors:** Bartosz Klebowski, Joanna Depciuch, Malgorzata Stec, Dawid Krzempek, Wiktor Komenda, Jarek Baran, Magdalena Parlinska-Wojtan

**Affiliations:** 1 Institute of Nuclear Physics Polish Academy of Sciences, 31-342 Krakow, Poland; bartosz.klebowski@gmail.com (B.K.); joannadepciuch@gmail.com (J.D.); dawid.krzempek@ifj.edu.pl (D.K.); wiktor.komenda@ifj.edu.pl (W.K.); 2 Department of Clinical Immunology, Jagiellonian University Medical College, 30-663 Krakow, Poland; malgorzata.stec@uj.edu.pl

**Keywords:** gold–platinum nanocauliflowers, radiosensitizers, proton therapy, MTS test, transmission electron microscopy, green chemistry, gallic acid

## Abstract

Enhancing the effectiveness of colorectal cancer treatment is highly desirable. Radiation-based anticancer therapy—such as proton therapy (PT)—can be used to shrink tumors before subsequent surgical intervention; therefore, improving the effectiveness of this treatment is crucial. The addition of noble metal nanoparticles (NPs), acting as radiosensitizers, increases the PT therapeutic effect. Thus, in this paper, the effect of novel, gold–platinum nanocauliflowers (AuPt NCs) on PT efficiency is determined. For this purpose, crystalline, 66-nm fancy shaped, bimetallic AuPt NCs were synthesized using green chemistry method. Then, physicochemical characterization of the obtained AuPt NCs by transmission electron microscopy (TEM), selected area electron diffraction (SAED), energy dispersive X-ray spectroscopy (EDS), and UV-Vis spectra measurements was carried out. Fully characterized AuPt NCs were placed into a cell culture of colon cancer cell lines (HCT116, SW480, and SW620) and a normal colon cell line (FHC) and subsequently subjected to proton irradiation with a total dose of 15 Gy. The 3-(4,5-dimethylthiazol-2-yl)-5-(3-carboxymethoxyphenyl)-2-(4-sulfophenyl)-2H-tetrazolium (MTS) test, performed after 18-h incubation of the irradiated cell culture with AuPt NCs, showed a significant reduction in cancer cell viability compared to normal cells. Thus, the radio-enhancing features of AuPt NCs indicate their potential application for the improvement in effectiveness of anticancer proton therapy.

## 1. Introduction

Malignant tumors are civilization-related diseases that more and more people suffer from every year worldwide. Among them, colorectal cancer is the third most common, and this type of cancer can be successfully cured at its early stage by surgical treatment [1]. Unfortunately, at more advanced stages, other methods such as radiation or chemotherapy alone or in combination with immunotherapy are recommended; however, their effectiveness is still limited [2,3,4]. Radiation-based anticancer therapy, such as proton radiotherapy, can be used in selective cases, especially before surgery (along with chemotherapy), to help shrink a tumor and to make it easier to remove or to enhance the effectiveness of immunotherapy [5,6]. The application of a proton beam enables precise irradiation of the tumor and, thus, saving normal tissues [7]. However, it is worth noting that the method of proton delivery (and at the same time, the precision of irradiation) depends on the region of the Bragg curve that is used for tumor irradiation. In the case of the Plateau region, an approximately constant value of relative biological effectiveness (RBE) = 1.1 is obtained. On the other hand, in the case of a Bragg peak, we get a high, rapidly changing RBE, making it difficult to determine the actual radiation dose [8]. In this regard, it is highly advisable to find a new tool, which will improve this type of neoadjuvant therapies. The help comes to us with so-called radiosensitizers [9].

Radiosensitizers, being low in toxicity, significantly improve the efficiency of radiation-based anticancer therapies such as X-ray radiation-, proton-, or photodynamic therapy [10,11,12]. Radiosensitizers are divided into several types: small molecular chemotherapeutics (e.g., gemcitabine), macromolecules (microRNA and proteins), or nanoparticles (NPs) [13]. The aforementioned radiosensitizers are aimed at improving the effectiveness of, e.g., proton therapy (PT), while reducing its side effects because the radiation dose used in this case can be much lower [14]. Currently, the application of NPs, especially noble metal NPs, for this purpose is highly promising.

Nanomedicine deals with the use of sub-micrometer materials not only as radiosensitizers but also as drug delivery systems or contrast agents in medicine [15]. It is associated with a number of undoubted advantages of such nanomaterials, which include their small size, large surface area, and stability [16,17]. Noble metal NPs, especially gold nanoparticles (Au NPs), which have fascinating abilities, ought to be highlighted in a special way. Their capacity for enhancement of photoelectric interaction at lower energy levels as well as the possibility of increasing the energy deposited in the tumor deserves attention [18]. Under the influence of the proton beam, Au NPs (and other high atomic number NPs) generate reactive oxygen species (ROS) and free radicals which are destructive to deoxyribonucleic acid (DNA), causing its breakage and, thus, cell death [12,19,20,21,22,23,24]. A similar effect, however with different powers, can be seen for other types of noble metal NPs, e.g., platinum (Pt NPs), gadolinium (Gd NPs), and silver (Ag NPs) [9,12]. 

The aim of this study was to establish the synthesis of novel, fancy-shaped bimetallic gold–platinum nanocauliflowers (AuPt NCs) with a highly developed surface area. This is of interest because there is limited information about the application of bimetallic NPs to support the effect of proton irradiation. AuPt NCs were prepared using a green chemistry, eco-friendly method, applying gallic acid as a “green” reducing agent. 

**Figure 1:**
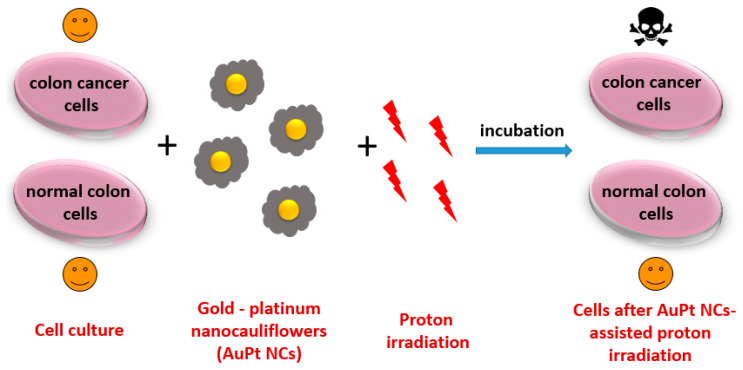
Overview scheme of the investigation procedure.

In this paper, we examined the radio-sensitizing effect of bimetallic 66-nm AuPt NCs on three cancer cell lines with different malignancy: HCT116, SW480, and SW620 as well as the normal colon cell line FHC. The efficiency of the PT supported by AuPt NCs was investigated by a cytotoxicity 3-(4,5-dimethylthiazol-2-yl)-5-(3-carboxymethoxyphenyl)-2-(4-sulfophenyl)-2H-tetrazolium (MTS) assay. Physicochemical characterization of AuPt NCs was also carried out. Transmission electron microscopy (TEM) served to assess the morphology of the obtained AuPt NCs. The chemical composition of as-prepared AuPt NCs was analyzed by energy dispersive X-ray spectroscopy (EDS). Selected area electron diffraction (SAED) was used to examine the crystal structure of AuPt NCs. The UV-Vis spectrum was also measured. An overview scheme of the investigation procedure is presented in Figure 1.

## 2. Results and Discussion

### 2.1. Mechanism of AuPt NCs (Gold-Platinum Nanocauliflowers) Synthesis

In the studies presented, we obtained gold–platinum NPs with a novel shape resembling cauliflowers. They were prepared by chemical reduction of gold and platinum (HAuCl_4_ and H_2_PtCl_6_, respectively) precursors using gallic acid, which is both a reducing agent and a stabilizer preventing agglomeration of these NPs. Gallic acid, being an environmentally friendly “green” reagent, is also relatively cheap. Moreover, gallic acid exhibits a broad spectrum of activity when it comes to reducing various metal precursors, which allows it to be used in the synthesis of numerous monometallic NPs as well as multicomponent nanocomplexes [25,26,27,28,29]. By controlling the reaction parameters (e.g., temperature or polyvinylpyrrolidone addition), it is possible to obtain nanoparticles with various sizes [30]. Such reducing properties of gallic acid are due to the fact that it consists of an aromatic ring containing a carboxyl and hydroxyl group. These groups form chelating rings with metal ions, which are oxidized to benzoquinones by exposure to air or electrophilic ions [31].

**Figure 2:**
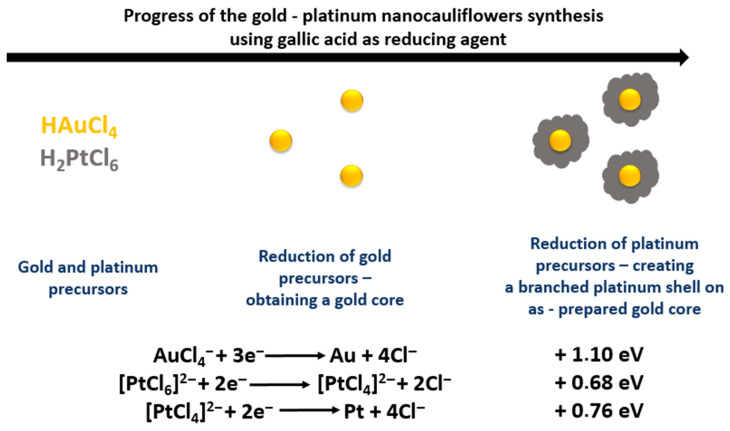
Synthesis mechanism of AuPt nanocauliflowers (NCs) using gallic acid.

The proposed reaction mechanism of the AuPt NCs synthesis is presented in Figure 2.

A color change of the solution to brown was observed directly after the addition of gallic acid to the reaction mixture containing gold and platinum precursors. The very fast formation of AuPt NCs was due to the significant difference in standard redox potentials of the two metal precursors [30]. The individual ions were reduced to the corresponding metals according to the reactions presented in Figure 2. In turn, gallic acid was transformed into its oxidized form [32]. This reaction took place in two steps, which proceed very quickly one after the other. In the first step, gallic acid reduced gold ions, as they have a higher standard redox potential. Thus, Au NPs were formed as cores on which the platinum nanoparticles (Pt NPs) could later be deposited. The second stage of AuPt NC synthesis consisted of two-stage reduction of platinum ions—initially to [PtCl_4_]^2−^ ions and finally to metallic platinum, which was deposited on the nanogold surface. It is worth noticing that, due to the fact that these reactions took place almost simultaneously, only the final brown color corresponding to AuPt NCs was observed in the reaction mixture. On the other hand, we did not observe the red color of the solution corresponding to formation of Au NPs with a size of about 20 nm [33]. The formation of a porous platinum shell was ensured by the addition of excess platinum precursors to the reaction mixture. 

### 2.2. Physicochemical Characterization of AuPt NCs

**Figure 3:**
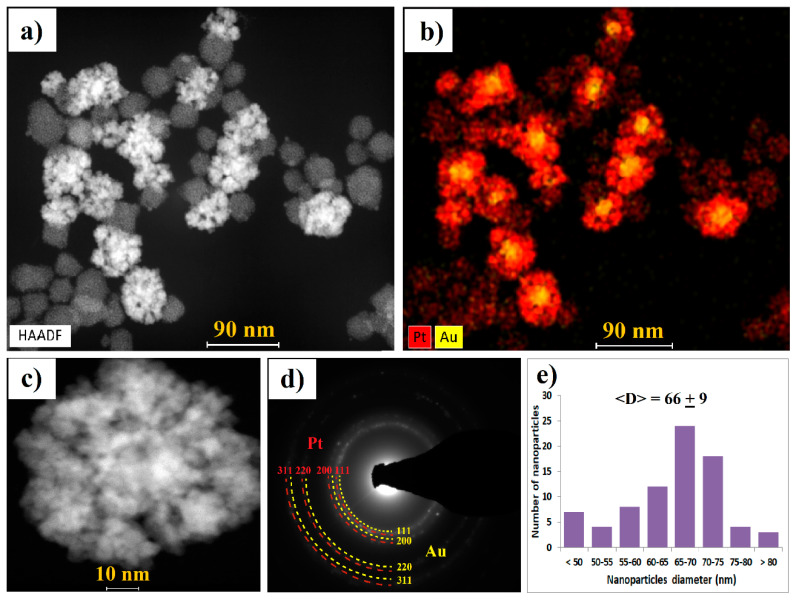
Morphology of AuPt NCs: (**a**) scanning transmission electron microscopy (STEM) High-angle annular dark-field detector (HAADF) overview image, (**b**) energy dispersive X-ray spectroscopy (EDS) distribution map of gold (yellow) and platinum (red) in AuPt NCs, (**c**) STEM HAADF higher magnification image, (**d**) corresponding selected area electron diffraction (SAED) pattern, and (**e**) size distribution of AuPt NCs.

Several techniques were used to fully characterize the obtained AuPt NCs. The morphology, chemical composition, and microstructure of AuPt NCs were determined by scanning transmission electron microscopy (STEM) with the High-angle annular dark-field detector (HAADF) detector, energy dispersive X-ray spectroscopy (EDS) maps, and the SAED patterns taken in the TEM mode (Figure 3a–d). Based on the STEM images, the size distribution of AuPt NCs was estimated (Figure 3e).

Reduction of gold and platinum precursors using gallic acid resulted in preparation of monodisperse AuPt NCs with an average size of 66 nm (Figure 3e). The size of the gold core was estimated to be around 20 nm. The shape of both Au NPs and Pt NPs was spherical. We observed that the platinum shell was composed of small, less than 5 nm Pt NPs agglomerated into larger nanostructures. On the STEM image (Figure 3a) and corresponding EDS map of AuPt NCs (Figure 3b, Figure S1), we noted the presence—in addition to AuPt NCs—of agglomerated Pt NPs consisting of smaller, approximately 2 nm Pt NPs without a gold core. These Pt NPs had weaker contrast due to the lower Z atomic number of platinum (Z = 78) than gold (Z = 79).

From the SAED pattern, it was found that the obtained AuPt NCs have a crystalline structure (sharp diffraction rings with bright spots were observed). The diffraction rings (Figure 3d) could be attributed to the (111), (200), (220), and (311) lattice planes of Au and Pt nanocrystals crystalized in the face-centered cubic structure [34]. 

**Figure 4:**
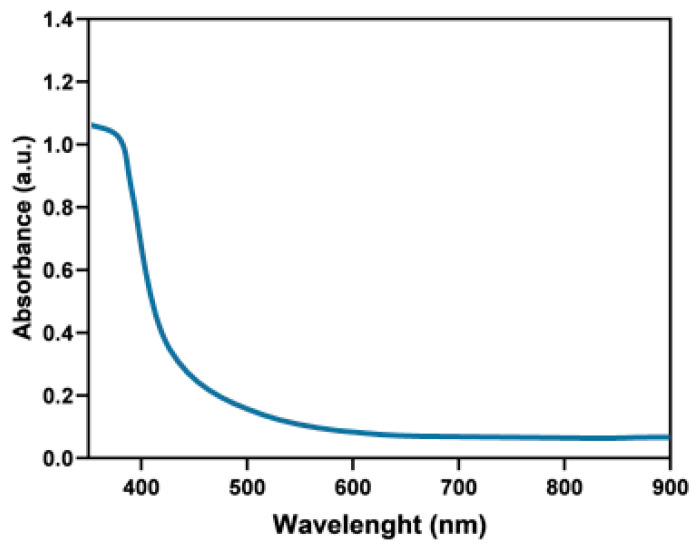
UV-Vis spectrum of AuPt NCs.

The UV-Vis measurements of AuPt NCs were also carried out (Figure 4). We did not detect any peak in the analyzed spectral range. Spherical Au NPs should have given an absorption peak in the visible light spectrum; however, we did not observe it [35]. This proved that the nanogold core was completely covered with Pt NPs, for which there was no absorption peak in the UV-Vis range [36]. Absence of any other peaks in this wavelength range indicated a lack of sample contamination.

### 2.3. Impact of AuPt NCs on Enhancement of the Proton Beam Irradiation Effect for Selected Cell Lines

The changes in the colon cancer and normal epithelium cells’ viability caused by AuPt NCs as well as by AuPt NC-assisted proton bream irradiation were determined using the MTS assay. The description of all the samples analyzed in this experiment is shown in Table 1. 

**Figure 5:**
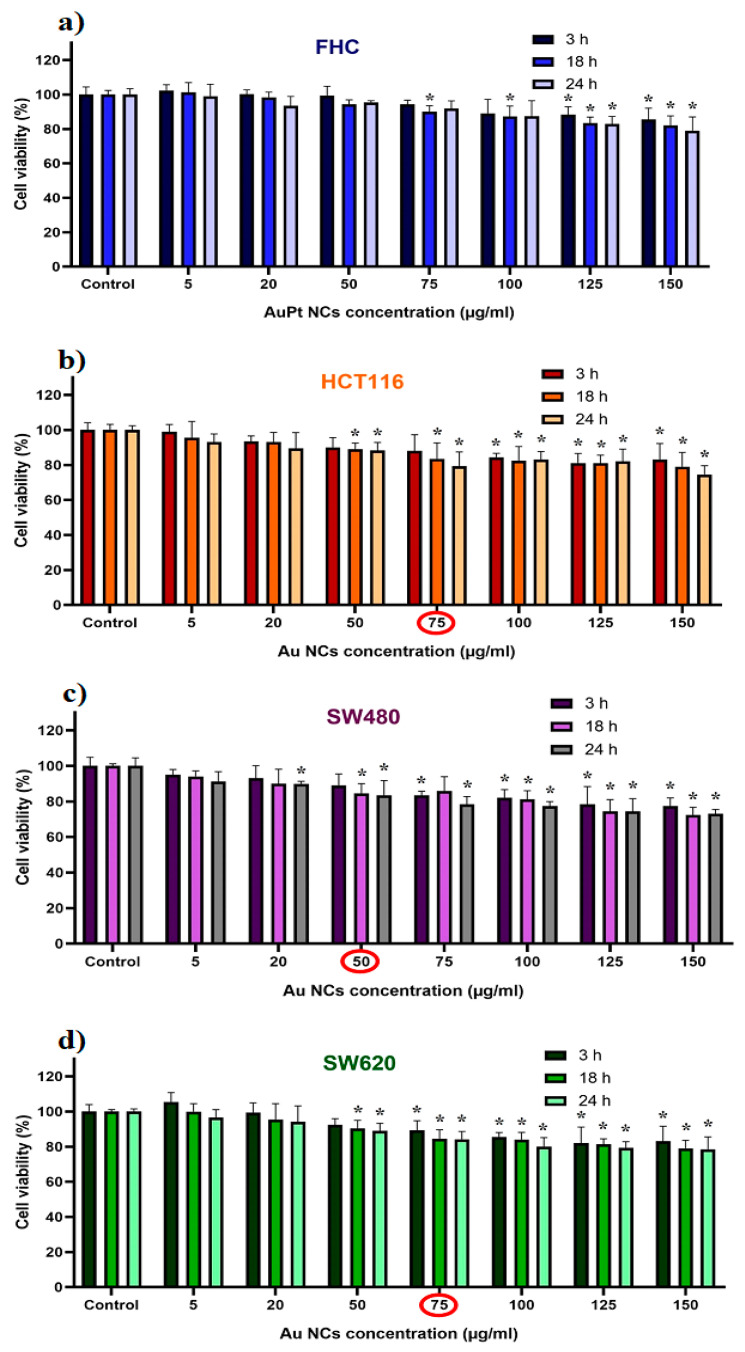
Cytotoxicity of AuPt NCs against (**a**) FHC, (**b**) HCT116, (**c**) SW480, and (**d**) SW620 cells after 3, 18, and 24 h of incubation: data were considered significant when * *p* < 0.05 versus control. The concentrations of HCT116, SW480, and SW620 cells considered optimal were marked in red.

Firstly, the cytotoxicity of AuPt NCs was determined. For this purpose, different concentrations of AuPt NCs were used in the cultures with cancer or normal cells at three different incubation times. The obtained results are presented in Figure 5.

The highest cytotoxicity of NCs—for all three incubation times—was noticed for the SW480 primary cancer cells. This is consistent with other reports—SW480 cells are generally more sensitive to some NPs or drugs than the SW620 or HCT116 cells [37,38,39]. The cytotoxicity of NPs depends not only on the metals used but also on their size, shape, or synthesis method [40,41]. Different concentrations of AuPt NCs were chosen for AuPt NC-assisted proton irradiation: 50 µL/mL for SW480 and 75 µL/mL for SW620 and HCT116 cells, respectively. These concentrations were considered as not causing a significant decrease in viability of these cells, i.e., less than 15% of cells were dead. Moreover, normal FHC cells were treated with both AuPt NCs concentrations to reliably compare the proton irradiation effect on the cancer and normal cells. There was no difference in cell viability for the 18 and 24 h incubation times, so further studies were carried out for 18 h of incubation of NPs with cells. It should be also mentioned that before AuPt NCs addition to the cell culture, the number of cells in the Burker chamber was counted to ensure approximately the same NP–cell ratios for all investigated cell lines.

**Figure 6:**
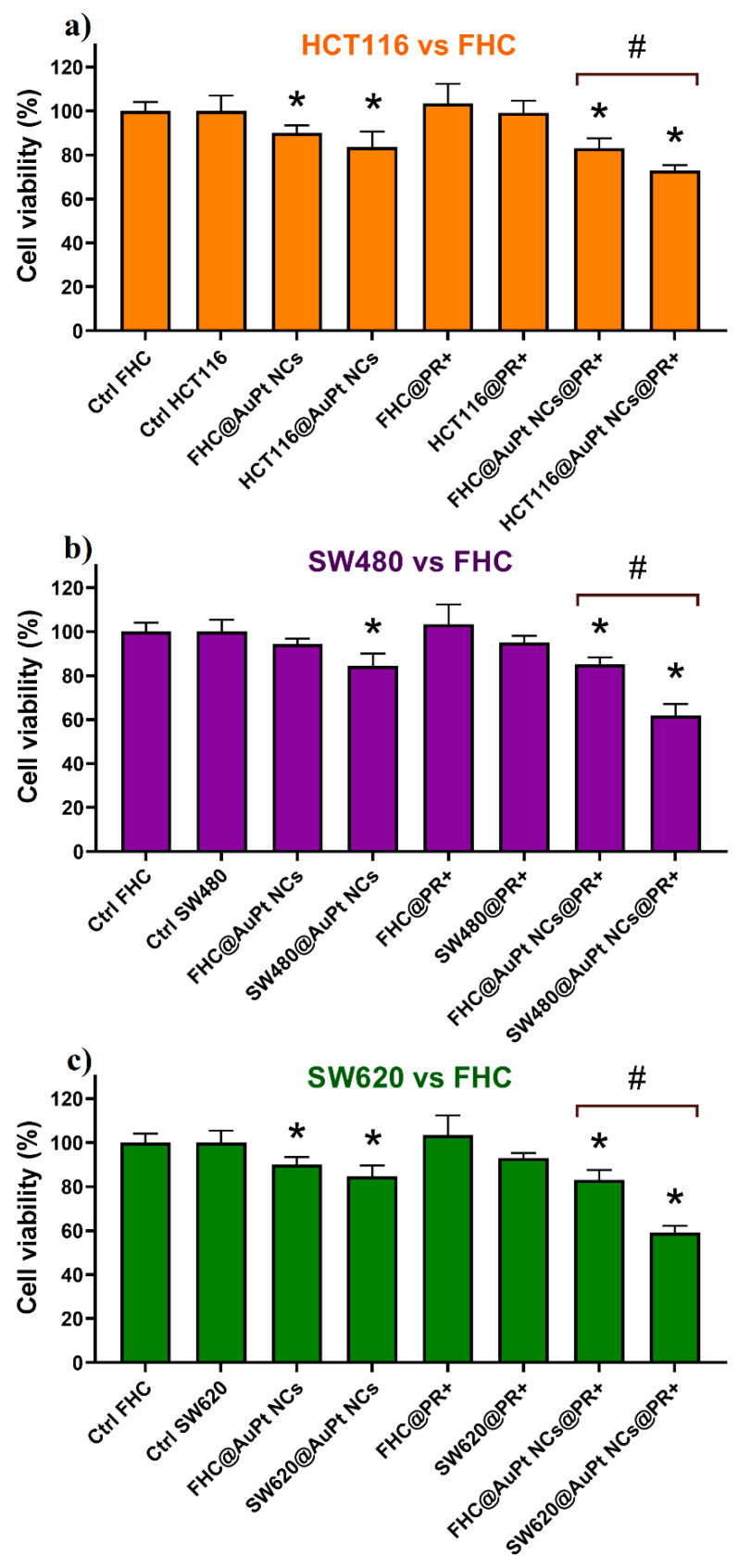
Viability of (**a**) HCT116, (**b**) SW480, and (**c**) SW620 cancer cells compared to FHC normal cells after the addition of AuPt NCs and proton irradiation of a total dose of 15 Gy combined with AuPt NCs: data were considered significant if * *p* < 0.05 versus control; # *p* < 0.05—statistically significant differences between the respective cancer line and the normal line.

In the next step, the effectiveness of AuPt NC-supported proton beam irradiation with a total irradiation dose of 15 Gy and 18 h incubation time after irradiation was investigated. The results are shown in Figure 6. The highest decrease in cell viability (93%) after irradiation with the proton beam (without the addition of AuPt NCs) was observed for the SW620 cells. In turn, FHC cells were less radiosensitive than both types of colon cancer cells, which is in line with the literature [42]. AuPt NC-assisted proton beam therapy turned out to be more effective than proton radiotherapy without the presence of NPs. It was observed that the addition of AuPt NCs, with a concentration which does not decrease cell viability by more than 15%, to the cell culture, significantly enhances the effect of irradiating cells with protons (Figure 6a–c). The highly developed surface of this type of bimetallic NP ensures large contact area of the cells with AuPt NCs, which results in generation of an increased amount of ROS destructive to cancer cells. A similar effect was observed when using porous Pt NPs as radiosensitizers in this type of anticancer therapy [43]. In Figure 6, statistically significant differences between the C@AuPt NCs@PR^+^ and Ctrl were observed. The addition of AuPt NCs to the cell culture followed by proton irradiation resulted in a mortality of approximately 16%, 26%, 38%, and 41% for FHC, HCT116, SW480, and SW620 cells, respectively. Among all studied cancer cell lines, the HCT116 cells are characterized by the lowest sensitivity to AuPt NC-assisted proton irradiation, which is confirmed by previous reports on the high malignance/metastasis potential of this cell line [44,45]. Moreover, there was a significant statistical difference in viability between normal FHC cells and cancer cells irradiated with protons and cultured with the same concentration of AuPt NCs. The negligible influence of this type of combined therapy on the normal cells is highly desirable from a therapeutic point of view. The presence of gallic acid, being a stabilizer of AuPt NCs, may influence such a selective action of this type of therapy on cancer. This compound has anti-inflammatory, antiallergic, antibacterial, and anticancer properties [32,46,47]. Several studies have shown that gallic acid has a selectively toxic effect on cancer cells, while saving normal ones [48,49,50]. Improving the selectivity of NPs towards cancer cells is particularly important for application of these nanomaterials in the clinic. For this purpose, it is desirable to biofunctionalize such NPs with some compounds increasing the cellular uptake by cancer cells. In the literature, there are reports on modification of nanoparticles with proteins (e.g., albumin) or tumor-specific antibodies, e.g., anti-Her2/neu or anti-epidermal growth factor receptor (EGFR) [51,52].

Summarizing, the results of our research indicate the high application potential of AuPt NCs in proton beam irradiation of human cancer. Further progress in this area may allow access to an effective tool to support also other radiation-based anticancer therapies, e.g., photon- or photodynamic therapy. 

## 3. Materials and Methods

### 3.1. Reagents and Chemicals

Hydrogen tetrachloroaurate trihydrate (HAuCl_4_ × 3H_2_O), hydrogen hexachloroplatinate hexahydrate (H_2_PtCl_6_ × 6H_2_O), and gallic acid were purchased from Sigma-Aldrich (Saint Louis, MO, USA). Chemical reagents were used without additional purification or modification.

### 3.2. AuPt NCs Synthesis

AuPt NCs were synthesized by green chemistry method using gallic acid acting simultaneously as a stabilizer and reducing agent. One hundred µL of HAuCl_4_ and 400 µL of H_2_PtCl_6_ aqueous solutions (10^−2^ M) were mixed in 17.5 mL distilled water in a round-bottom flask. The reaction mixture was heated until boiling on a magnetic stirrer (300 rpm), and then 2 mL of 5 × 10^−3^ M freshly prepared gallic acid solution was added. The reaction was carried out for 30 min, observing color change of the solution from colorless to brown, which indicates the formation of AuPt NCs.

### 3.3. TEM Characterization

Scanning transmission electron microscopy (STEM) combined with the high-angle annular dark-field detector (HAADF) working in conventional and high-resolution mode served as a tool to evaluate the morphology of prepared AuPt NCs. SAED patterns were taken in the TEM mode. All these measurements were performed on an aberration-corrected FEI Titan electron microscope operating at 300 kV equipped with an FEG (field emission gun) cathode. EDS mappings were done using a FEI Talos TEM operating at 200 kV equipped with an FEG cathode and four in-column EDS detectors (Super EDS system) The particle size distribution was evaluated based on STEM images taken from different areas of the TEM grids, which showed that the morphology of the AuPt NCs is the same in the whole analyzed sample (Figure S2). The AuPt NCs were measured and analyzed using TIA Software. The diameter of about 100 AuPt NCs was measured as a distance between the two most distant points of these NPs. 

### 3.4. UV-Vis Spectroscopy

The UV-Vis measurements were performed using a UV-2600 instrument from Shimadzu. The resolution was chosen to be 1 nm, and the sampling interval was 1. The volume of each sample was 1 mL. The spectral range was from 200 nm to 900 nm.

### 3.5. Cell Culture

Colon cancer cell lines (SW480 and SW62) were obtained due to the courtesy of Prof. Caroline Dive, Paterson Institute for Cancer Research, University of Manchester. Human colon carcinoma cell line HCT116 and fetal colon cell line FHC (CRL-1831) were obtained from the American Type Culture Collection (ATCC, Manassas, VA, USA) and maintained according to the ATCC’s instructions. HCT116 cells were cultured in McCoy’s 5A medium (Gibco, Paisley, UK). SW480 and SW620 cells were cultured in DMEM (Dulbecco’s modified eagle medium) with high glucose (Corning, NY, USA). FHC cells were cultured in DMEM/F12 medium (Gibco) supplemented with 25 mM HEPES (4-(2-hydroxyethyl)-1-piperazineethanesulfonic acid), 10 ng/mL cholera toxin, 0.005 mg/mL insulin, 0.005 mg/mL transferrin, and 100 ng/mL hydrocortisone. All media were supplemented with 10% fetal bovine serum (FBS, Gibco) and ciprofloxacin (10 µg/mL). The cells were cultured by biweekly passages in a 37 °C humidified atmosphere with 5% CO_2_ and regularly tested for *Mycoplasma* sp. contamination.

### 3.6. Proton Irradiation and Dosimetry

Irradiations were performed in the Cyclotron Centre Bronowice, Institute of Nuclear Physics Polish Academy of Sciences, Krakow, Poland. The proton therapy system was installed in the Centre consists of a IBA proteus C-235 cyclotron (IBA PT, Louvain-la-Neuve, Belgium) and two gantries equipped with scanning nozzles. In pencil beam scanning (PBS) techniques, a narrow proton beam is deflected in two perpendicular directions, delivering the dose point by point to the whole target volume. Irradiations were performed using a monoenergetic field with an energy of 225 MeV and dimensions of 20 cm × 20 cm. The cells were irradiated at 1.1 cm water equivalent depth with a dose of 15 Gy. The gantry was set at 180°, meaning that the beam was directed from the bottom to the top. The preparation of the experiment included dosimetry measurements performed with a Markus type ionization chamber calibrated in terms of dose absorbed to water. A radiation dose of 15 Gy was experimentally selected for our research as nontoxic to the cell lines used. After proton irradiation, each cell line was incubated for 18 h and then cell viability was assessed by MTS assay. 

### 3.7. MTS Viability Assay

Cytotoxic activity of AuPt NCs against normal and cancer cells was determined using 3-(4,5-dimethylthiazol-2-yl)-5-(3-carboxymethoxyphenyl)-2-(4-sulfophenyl)-2H-tetrazolium (MTS) assay (CellTiter 96^®^ Aqueous One Solution Cell Proliferation Assay, Promega, Madison, WI). Briefly, the cells were cultured in flat-bottom 96-well plates (Sarstedt, Numbrecht, Germany) at a density of 1 × 104/well in DMEM medium containing 10% FBS. After 48 h, 20 μL of AuPt NC solutions with different concentrations was added to the 100 μL medium with cells, giving a final concentration of AuPt NCs in the medium in the range between 5–150 µg/mL. After incubation periods of 3, 18, and 24 h, 20 µL of MTS dye solution was added per well. The quantity of formazan product, directly proportional to the number of living cells in culture, was detected by absorbance measurements at 490 nm with a 96-well plate reader (Spark^®^ Tecan, Mannedorf, Switzerland). For further AuPt NC-assisted proton irradiation studies, a maximum concentration which did not cause a decrease in cell survival by more than 15% after 18 h incubation was selected (50 µg/mL for SW480 and 75 µg/mL for SW620 and HCT116 cells). As for the FHC cell line (control), these cells were treated with both (50 and 75 µg/mL) AuPt NC concentrations allowing reliable comparison of the effect of proton irradiation on normal and colon cancer cells.

### 3.8. Statistical Analysis of Cell Viability Data

The obtained MTS assay results are represented as the means ± SEM (standard error of the mean). The experimental data were analyzed by one-way analysis of variance (ANOVA) followed by post hoc Tukey test. *p* value < 0.05 was considered statistically significant. The data were analyzed and presented graphically using GraphPad Prism 8 Software.

## 4. Conclusions

In this work, we have shown a synergistic effect of 66 nm AuPt NCs and proton irradiation towards the destruction of colon cancer cells. Synthesized by green chemistry and fully characterized by physicochemical methods, AuPt NCs showed a highly developed active surface, which allowed them to have a larger contact area with the cells. This fact as well as the presence of gallic acid—having strong anticancer properties—in the structure of AuPt NCs gave promising results. AuPt NC-assisted proton irradiation proved to be particularly effective in killing cancer cells (especially in SW480 and SW620 colon cancer cells, where a decrease in survival to approximately 60% was observed), while normal cells were only slightly affected (viability > 80%). Moreover, the application of green chemistry reagents for the production of NPs is favorable for ecology and ensures higher biocompatibility of such nanomaterials. Thus, the development of new and the improvement of already known metallic NPs in the future may contribute to finding the “golden mean” in the fight against various types of cancers.

## Figures and Tables

**Table 1:** Description of the investigated samples.

Sample	Name of Sample in the Manuscript
SW480, SW620, HCT116, and FHC cells without addition of AuPt nanocauliflowers (NCs) and proton irradiation (controls)	Ctrl
Cells cultured with AuPt (NCs)	C@AuPt NCs
Cells irradiated by proton beam	C@PR^+^
Cells cultured with AuPt NCs and irradiated by proton beam	C@AuPt NCs@PR^+^

## Supplementary Materials

The following are available online at https://www.mdpi.com/1422-0067/21/24/9610/s1, Figure S1: (A) STEM higher magnification image of AuPt NCs, EDS distribution maps of: (B) gold and platinum in the AuPt NCs, (C) gold (yellow) and (D) platinum (red), separately, Figure S2: Determination of the size of AuPt NCs as the distance between two extreme points of these NPs based on STEM HAADF images.

## Abbreviations

Ag NPs	Silver nanoparticles
AuPt NCs	Gold–platinum nanocauliflowers
DMEM	Dulbecco’s modified eagle medium
EDS	Energy dispersive X-ray spectroscopy
EGFR	Epidermal growth factor receptor
FBS	Fetal bovine serum
FEG	Field emission gun
Gd NPs	Gadolinium nanoparticles
HAADF	High-angle annular dark-field detector
HEPES	4-(2-hydroxyethyl)-1-piperazineethanesulfonic acid
MTS	3-(4,5-dimethylthiazol-2-yl)-5-(3-carboxymethoxyphenyl)-2-(4-sulfophenyl)-2H-tetrazolium
PBS	Pencil beam scanning
PT	Proton therapy
Pt NPs	Platinum nanoparticles
RBE	Relative biological effectiveness
ROS	Reactive oxygen species
SAED	Selected area electron diffraction
STEM	Scanning transmission electron microscopy
