# Supplementary material for: Fancy-Shaped Gold–Platinum Nanocauliflowers for Improved Proton Irradiation Effect on Colon Cancer Cells
Source: Int J Mol Sci. 2020 Dec 17;21(24):9610. doi: 10.3390/ijms21249610 (PMC7766784; doi:10.3390/ijms21249610)
Supplement: Supplementary file 1 [file ijms-21-09610-s001.pdf]

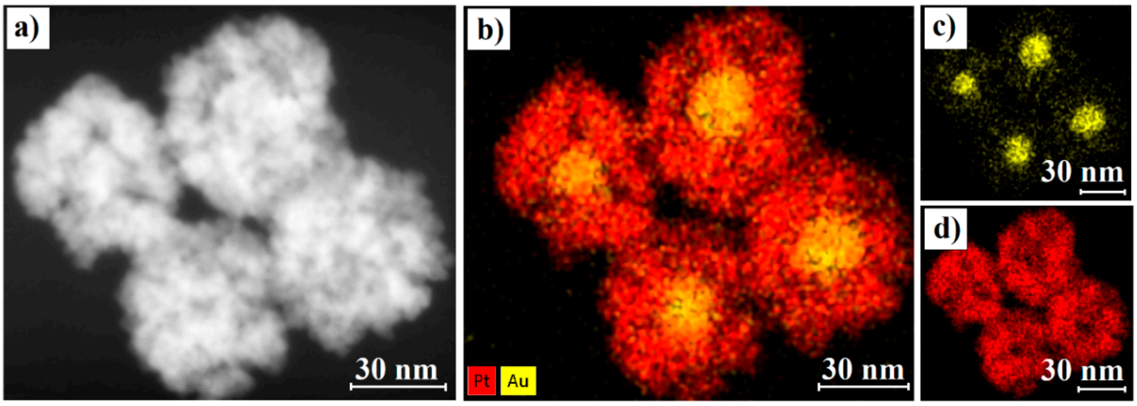

Figure S1. (A) STEM higher magnification image of AuPt NCs, EDS distribution maps of: **(B)** gold and platinum in the AuPt NCs, **(C)** gold (yellow) **(D)** platinum (red), separately.

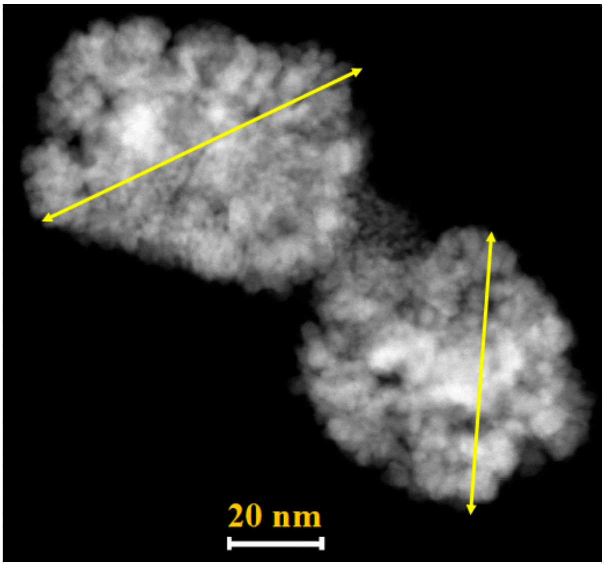

Figure S2. Determination of the size of AuPt NCs as the distance between two extreme points of these NPs based on STEM HAADF images.
